# Influence of Design Parameters on Implant Abutment Performance: A Scoping Review

**DOI:** 10.3390/jfb16090342

**Published:** 2025-09-11

**Authors:** Vladimir Prpic, Petar Kosec, Stanko Skec, Amir Catic

**Affiliations:** 1 Department of Fixed Prosthodontics, School of Dental Medicine, University of Zagreb, Gunduliceva 5, 10000 Zagreb, Croatia; 2 Department of Design and Product Development, Faculty of Mechanical Engineering and Naval Architecture, University of Zagreb, Ivana Lucica 5, 10000 Zagreb, Croatia; petar.kosec@fsb.hr (P.K.); stanko.skec@fsb.hr (S.S.); 3 Department of Fixed Prosthodontics, School of Dental Medicine, University of Zagreb, Clinical Hospital Centre Zagreb, Gunduliceva 5, 10000 Zagreb, Croatia; catic@sfzg.hr

**Keywords:** transmucosal height, prosthodontic height, implant abutment, generative design, prosthodontics

## Abstract

Implant abutments serve as the critical interface between dental implants and prosthodontic restorations, playing a central role in the functional and aesthetic success of implant-supported rehabilitations. With the development of CAD/CAM technologies, dental implantology has experienced a significant transformation. The latest and still unexplored field of science includes an advanced algorithm-driven process known as generative design. Generally, generative design is used to investigate a broad spectrum of design alternatives that satisfy predetermined criteria established by a designer. Prior to the application of generative design, it is essential to define the key parameters that influence the optimization of the designed object’s configuration, such as a hybrid implant abutment. A bibliographic search was performed using PubMed and Scopus databases to identify relevant studies published up to 1 July 2025. Studies that investigated transmucosal and prosthodontic height of implant abutments were selected for inclusion. Only 13 studies met inclusion criteria and were further analyzed. Included studies showed the importance of transmucosal and prosthodontic height of implant abutments in order to reduce possible complications. The review highlights the importance of optimizing the transmucosal and transgingival heights of implant abutments. For predictable biological and mechanical outcomes, both dimensions should be at least 2 mm. The obtained parameter values will be used to create the initial parametric model, which will then be utilized in the generative design process of hybrid implant abutments.

## 1. Introduction

The rapid advancement of digital technologies has significantly transformed diagnostics, treatment planning, and therapeutic procedures in dental medicine, most notably in the areas of implantology and implant prosthodontics. These innovations have introduced a broad range of new concepts, approaches, and workflows aimed to enhance clinical predictability and procedural efficiency while also minimizing the likelihood of complications [1,2,3,4]. Dental implants represent a widely accepted and clinically validated solution for the replacement of extracted or missing teeth with a high success rate of 90% [5,6,7]. Implant therapy with two-piece implant systems involves a multi-step process, integrating both surgical and prosthodontic components. The surgical component includes a dental implant, mostly made of titanium, a biocompatible material that is inserted into the alveolar bone of the mandible or maxilla [8]. Implant abutment serves as the critical connecting component in implant prosthodontic therapy, linking the implant to the prosthodontic restoration [9,10]. Choosing the right implant abutment is crucial for optimal function, aesthetics, and long-term success with dental implants [11].

Stock implant abutments offer several advantages, including low cost, reliable engagement with the implant body, and the capability to be prepared either intraorally or extraorally. However, stock abutments also have certain limitations, such as extended preparation time, particularly in cases of mispositioned dental implants, since they do not conform well to the anatomical complexities of the gingival contour [12]. Inadequate adaptation of the implant abutment to the gingival margin may lead to the retention of excess cement in the peri-implant tissues during the cementation process. Thus, cement in the peri-implant sulcus can result in inflammation of soft and hard tissues surrounding dental implants [13]. Stock implant abutments are commonly made from two different types of materials, depending on the clinical situation in terms of strength, function, and appearance. Titanium abutments are most frequently used because of their well-documented biological and mechanical benefits. Conversely, the grayish discoloration beneath the peri-implant mucosa fail to meet the optical and aesthetic requirements for implant-supported restorations [14,15]. Zirconia abutments have suitable optical characteristics [14] but some challenges have been observed with the clinical use of zirconia abutments, such as risk of fracture and wear on the titanium implant where it connects with the zirconia [16]. Nevertheless, stock abutments do not always offer enough options in terms of size, angulation, and emergence profile, which can make it challenging to fully adapt them [17].

Peri-implantitis represents one of the most prevalent biological complications associated with dental implants, with epidemiological data indicating that approximately 20% of patients are likely to develop the condition over time. Despite extensive research, clinical management of peri-implantitis remains complex and continues to present significant therapeutic challenges [18]. A certain number of mechanisms have been used to explain the beneficial effect of higher abutments on marginal bone levels. Firstly, higher abutments provide increased space for the establishment of the supracrestal soft tissue attachment. Secondly, by positioning the abutment–crown interface more coronally, higher abutments distance the microgap from the crestal bone [18,19]. Microgaps are strongly associated with the extent of peri-implant bacterial colonization, which may compromise the sealing capacity of the peri-implant mucosa and thereby increase the risk of peri-implantitis as well as mechanical complications [20]. The application of digital technologies allows the fabrication of prosthetic components with high precision, thereby minimizing manufacturing errors and reducing the incidence of microgaps [21].

With the advancement of CAD/CAM (Computer-Aided Design/Computer-Aided Manufacturing) technology, the fabrication of implant abutments has increasingly been carried out by usage of this digital approach. The advantages include fewer manual steps, enabling a simplified production process and offering high predictability. Additionally, digitally designed and manufactured implant abutments can be customized to each specific case, offering advantages like the capability to be fabricated from various materials, most notably titanium and zirconia [22]. A novel method for achieving both aesthetics and strength in implant dentistry is the hybrid-abutment approach [23,24]. Hybrid implant abutments combine the benefits of both zirconia and titanium implant abutments, including improved aesthetics, optimal biological response, and superior mechanical properties, without negatively effecting the implant–abutment interface [24]. Appropriate adhesive bonding procedures between titanium and zirconia components of a hybrid implant abutment is essential to prevent retention loss of a prosthodontic restoration [25]. Nowadays, hybrid implant abutments are most frequently fabricated through digital workflow which requires a digital impression which can be obtained either directly from the oral cavity by using a scan body or indirectly from a cast model. A suitable titanium insert can be selected based on a specific implant system, while CAD/CAM software is utilized to design the zirconia component of hybrid implant abutment [24]. CAD/CAM abutments are designed digitally by the clinician or technician [22], relying on their input to shape and fit the component, whereas generative design uses algorithms to automatically create optimized geometries [26,27]. In CAD/CAM, the final design depends largely on the operator’s experience, while generative design explores multiple possibilities based on mechanical, biological, and aesthetic factors. CAD/CAM provides predictable and standardized results, minimizing errors and microgaps, but it does not inherently optimize performance. Generative design, on the other hand, can produce patient-specific abutments with unique shapes that improve stress distribution and support healthier peri-implant tissues. While CAD/CAM focuses on accurately reproducing a planned design, generative design emphasizes solutions tailored to the individual needs of each case. Together, these approaches show how digital technology is evolving in implant prosthodontics, combining precision with performance-focused innovation.

However, despite the increasing popularity of hybrid abutments, the current design process remains largely manual and highly dependent on the clinician’s or technician’s experience. This is particularly challenging given the complex interplay of mechanical and aesthetic requirements inherent in hybrid configurations. One of the most critical issues is the structural vulnerability of the zirconia component, which is prone to fracture under functional loading. Such failures not only compromise the prosthodontic restoration but may also require redesigning both the zirconia and titanium parts to restore function and stability. As a result, designing hybrid abutments involves balancing multiple interdependent factors such as structural integrity, aesthetics, fit, and adhesive integrity, making it challenging to achieve consistent, optimal outcomes. This highlights the need for a more systematic design approach that can guide the user through these competing priorities and provide performance-based design support.

Generative design is a computer-based design approach where a designer sets the key parameters, like material type, function, anatomy, and aesthetics, and the software takes over from there, automatically generating a range of optimized design options. Unlike traditional CAD, where a designer manually creates the shape, generative design lets the system explore multiple possibilities and propose the best solutions based on a given criteria [26,27]. The range of parameter values must be determined prior to the design process to optimize the outcome and ensure the most effective performance of the final object. In implant prosthodontics, generative design allows exploration of a wide range of different solutions to identify the optimal configuration of a given structure (e.g., hybrid implant abutment). Published data regarding standardized requirements for optimal design of hybrid implant abutments are scarce and all decisions are made based on prior experience and case-specific considerations. Accordingly, the broad implementation of generative design within the context of implant prosthodontics is not feasible.

The purpose of the present study was to conduct a literature review on the transmucosal and prosthodontic height of implant abutments to determine their optimal dimensions. The study focused on defining the key preparatory steps required for the effective implementation of generative design in implant prosthodontics.

## 2. Materials and Methods

**Figure 1:**
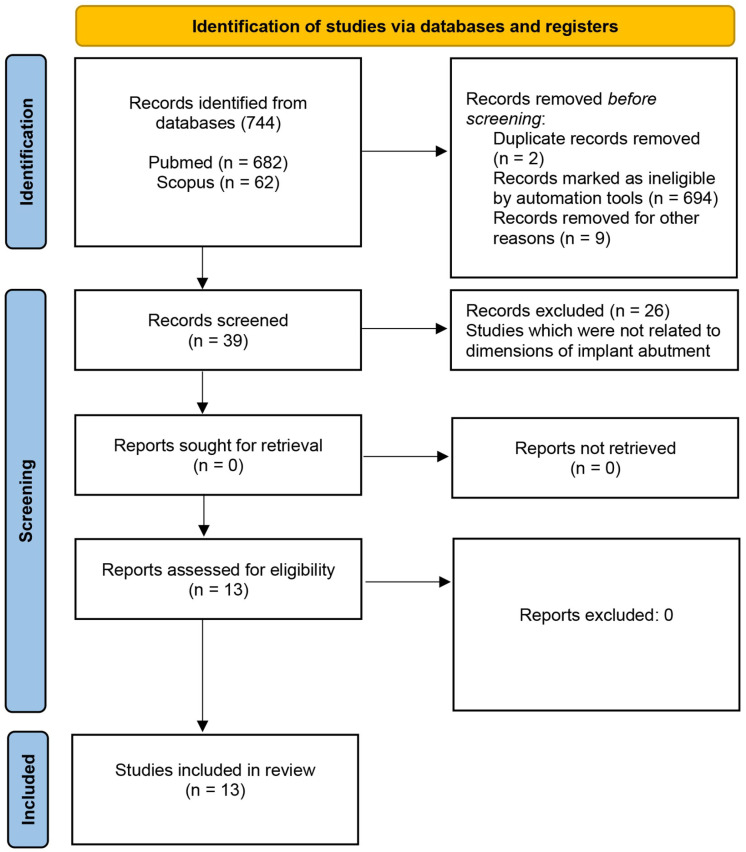
PRISMA-based flowchart illustrating the identification, screening, and selection process of the records during the search.

The scoping review was reported in accordance with the PRISMA 2020 (Preferred Reporting Items for Systematic Reviews and Meta-Analyses) guidelines [28] and was registered in the Open Science Framework (OSF) public registry (Registration DOI: https://doi.org/10.17605/OSF.IO/WNSKQ). A comprehensive literature search was conducted to identify all relevant studies addressing the primary purpose of the study. Studies that investigated transmucosal height of implant abutment and prosthodontic height of implant abutment in the last five years have been included in the literature review. The last five years have been selected to capture the most recent studies in the review and to ensure the inclusion of the most current and accurate evidence. This timeframe allows the analysis to reflect the latest advances and trends in the field. The exclusion criteria encompassed case series, editorials, and interview articles, as well as studies which were not written in the English language. A bibliographic search was performed across the PubMed and Scopus databases to identify relevant studies published up to 1 July 2025. Inclusion criteria comprised full-text studies published in the English language which were conducted on a human model. The search keywords were “transmucosal height of implant abutment” and “prosthodontic height of implant abutment”. The total number of studies included in the review is illustrated in a PRISMA-based flow diagram (Figure 1). Two experienced reviewers (V.P. and P.K.) independently assessed the titles of all retrieved studies based on predefined inclusion criteria. Subsequently, the abstracts of the selected studies were screened, and those deemed relevant were subjected to full-text evaluation. Inclusion of each study in the final analysis required consensus between both primary reviewers. In cases of disagreement or uncertainty, two additional reviewers (S.S. and A.C.) were consulted to reach the solution. The data extraction protocol encompassed the following parameters: authors and year of publication, study title, type of the study, sample size, and conclusion of the study (Table 1 and Table 2).

## 3. Results

Initially, a total of 744 studies were identified through the database search. Duplicates were removed and an automation tool was employed to remove ineligible studies which did not fulfill inclusion criteria. Thirty-nine studies entered the screening process. In the end, 13 studies fulfilled inclusion criteria and were further analyzed. Figure 1 shows a PRISMA (Preferred Reporting Items for Systematic Reviews and Meta-Analyses)-based flowchart illustrating the identification, screening, and selection process of the records during the search. Three out of thirteen studies were related to transmucosal height of implant abutment, while the rest of them were associated with prosthodontic height of implant abutments. Three studies that met inclusion criteria regarding transmucosal height of implant abutment include “Combined Effect of Abutment Height and Restoration Emergence Angle on Peri-Implant Bone Loss Progression: A Retrospective Analysis” by Misch et al. [29]; “Limited marginal bone loss in implant-supported fixed full-arch rehabilitations after 5 years of follow-up” by Galindo-Moreno et al. [30]; “Effects of Different Patient and Prosthetic Variables on Marginal Bone Behavior in Dental Implants: A Clinical Retrospective Study” by Gehrke et al. [31]. All aforementioned studies investigated the influence between transmucosal height of implant abutments and marginal bone loss and concluded that implant abutments should have height above 2 mm to reduce bone loss and to minimize the risk of peri-implantitis. Table 1 provides a synthesized summary of the data extracted from the studies included in the analysis. Studies which were associated with prosthodontic height of implant abutments are listed in Table 2. Six studies [32,33,34,35,36,37] investigated the influence of prosthodontic abutment height on marginal bone loss. The findings consistently indicate that a minimum prosthodontic implant abutment height of 2 mm is recommended to minimize marginal bone resorption around dental implants. The remaining four studies [38,39,40,41] focused on distinct aspects related to implant abutments: crown retention on implant abutments, the influence of abutment heights on screw loosening, the effect of abutment height on the pull-off force of crowns, and the fracture strength analysis of implant abutments, respectively. Retention of restorations was enhanced when longer implant abutments were utilized [38]. The height of the implant abutment has been shown to influence screw loosening [41] and long-term stability of restorations as well [40]. Only one study [39] comparing pull-off forces of cement-retained implant-supported single crowns on stock titanium abutments showed lesser influence of implant abutment height when compared to cement. From all of the above, the importance of optimal implant abutment dimensions becomes evident.

## 4. Discussion

Before applying generative design in implant prosthodontics, it is important to go through a careful parametrization process. This means identifying the biomechanical factors that will guide the design. This approach ensures that the generative design process remains within clinically relevant boundaries, producing outcomes that are not only anatomically accurate but also functionally appropriate for intraoral conditions. Parametrization acts as a foundation, helping the system understand what is important for each individual case. Without parametrization, the designs may not meet the practical needs of dental treatment. Therefore, a comprehensive review was conducted to collect and analyze the data related to the dimensional characteristics of implant abutments (transmucosal and prosthodontic height).

A review of three studies [29,30,31] (Table 1) that focused on transmucosal height of implant abutment showed coherent results. Misch et al. [29] analyzed the influence of transmucosal abutment height on marginal bone loss around bone-level implants. Marginal bone levels were assessed at two time intervals: 12–18 months post-crown placement (T0) and more than one year after the T0 radiograph (T1), on both mesial and distal aspects of the implant. The change in bone loss was determined as the difference between the measurements obtained at T0 and T1. Two different transmucosal abutment heights (<2 mm and >2 mm) were evaluated and it was concluded that abutments with a transmucosal height greater than 2 mm were significantly associated with a reduced incidence of peri-implantitis and marginal bone loss. A possible reason for this common observation is that short implant abutments do not leave enough vertical space for proper supracrestal tissue formation. This can lead to increased marginal bone loss, exposure of the implant’s rough surface, and even the implant threads, creating areas where plaque can accumulate and increasing the risk of peri-implantitis [29]. Galindo-Moreno et al. [30] investigated the influence of 4 different transmucosal heights of implant abutments (1 mm, 2 mm, 4 mm, and 6 mm) on marginal bone loss. Marginal bone levels were assessed by importing panoramic radiographs in DICOM (Digital Imaging and Communications in Medicine) format into an image analysis platform. Measurements were taken from the implant shoulder to the most coronal aspect of the supporting crestal bone, with negative values assigned when the bone crest was positioned apically relative to the implant shoulder. The results of the study showed that marginal bone loss was higher in 1 mm implant abutments when in comparison with 2 mm, 4 mm, and 6 mm implant abutments. This finding demonstrates that implant abutment height has a significant influence on marginal bone loss, following the principle that greater abutment height is associated with reduced marginal bone loss. The results of these two studies [29,30] are in accordance with the third included study in which Gehrke et al. [31] examined marginal bone-level alterations based on transmucosal abutment height. The transmucosal height of the evaluated abutments was categorized into three groups: the first group comprised abutments with heights of 0.8 and 1.5 mm; second group comprised abutments of 2.5 mm; and third group comprised abutments with heights ≥ 3.5 mm (3.5, 4.5, and 5.5 mm). Measurements were performed on radiographs obtained at time points C1 (rehabilitation installation) and C2 (final follow-up), and the change in values was calculated as the difference between C2 and C1. The authors concluded that implant abutment height greater than 2.5 mm has been associated with reduced peri-implant marginal bone loss. The observed differences in marginal bone loss among the groups indicate that, when clinically feasible, taller abutments should be preferred [31]. According to the recent study [29], implants with a transmucosal abutment height greater than 2 mm were found to have about four times less risk of developing peri-implantitis. The reviewed studies underscore the importance of optimizing implant abutment dimensions, emphasizing the critical role of precise parameterization before the generative design process.

A review of ten studies [32,33,34,35,36,37,38,39,40,41] (Table 2) dealing with prosthodontic height of implant abutment highlights its significant impact on soft tissue health, prosthodontic stability, and long-term implant success. Six studies [32,33,34,35,36,37] investigated the influence on marginal bone loss and showed congruent results. Studies showed that an abutment height of no less than 2 mm is recommended for optimal clinical outcomes. The impact of short abutments (<2 mm) on marginal bone loss may be attributed to two factors. Firstly, shorter abutments (<2 mm) are associated with reduced supracrestal tissue height and biological width, which may compromise the protective barrier around the initial bone-to-implant contact. Secondly, a shorter abutment (<2 mm) reduces the vertical distance between the implant shoulder and the prosthodontic interface, thereby positioning the oral microbiota in closer proximity to the first bone-to-implant contact, potentially facilitating bacterial infiltration and subsequent bone resorption [32].

One study [38] emphasized the impact of titanium-base (Ti-base) height on the retentive properties of hybrid abutment crowns. Two different implant abutment heights of 4 mm and 7 mm were tested. The 7 mm Ti-base showed noticeably stronger retention compared to the 4 mm Ti-base, regardless of the crown material used. The retention of fixed restoration is closely related to the total surface area of the implant abutment. Since all abutments in the analyzed study [38] had the same diameter, the surface area was mainly determined by their height. This means the longer abutments had more surface area than the shorter ones, which likely explains why they showed better retention. In conclusion the surface area of the abutment can be approximated by the product of its diameter and height [38]. The importance of the optimal abutment height is noticeable.

In a recent study [41], the authors compared the influence of different types (stock, cast, and milled) of abutments with standardized heights of 4 mm, 7 mm, and 10 mm on a screw loosening. Abutments with a 4 mm height demonstrated superior performance in maintaining screw torque compared to those of 7 mm and 10 mm heights [41]. Accordingly, height of abutment presents an essential factor which can affect the long-term success and mechanical stability of implant-supported restorations [41].

A study by Müller et al. [39] evaluated and compared the pull-off forces of cement-retained zirconia-reinforced lithium silicate single crowns placed on titanium abutments, focusing on how different abutment heights and types of implant cement affect retention. Abutment heights of 3 mm and 5 mm were utilized in the study [39]. The influence of abutment height on pull-off forces was found to be limited when compared to cements [39]. However, abutment height plays an important role in routine clinical practice.

Song et al. [40] examined fracture strength by adjusting the height (1 mm, 3 mm, and 5 mm) of the titanium insert. The goal was to determine which titanium insert height offers the best mechanical stability. The results of the study showed that the axial height of the titanium insert should be designed to be at least 3 mm [40]. A titanium insert with an axial height of 1 mm appears clinically inadequate due to its insufficient mechanical strength [40].

Choosing and adapting optimal implant abutment parameters clearly plays a key role in both biological response and technical performance of a restoration. It can make a real difference in how well the implant integrates with the bone, how stable the surrounding soft tissue remains, and how the restoration holds up over time. With modern tools like CAD/CAM and generative design, it is now possible to create solutions that are better tailored to each patient, leading to more reliable and long-lasting results.

Apart from the transmucosal and prosthodontic implant abutment heights, it is essential to define additional parameters before establishing the initial parametric model, to ensure optimal biomechanical performance, patient-specific adaptation, and the long-term clinical success of the implant-supported restoration. A careful analysis of additional factors should encompass both mechanical factors (implant abutment angulation, load distribution, and type of restoration) and biological factors (soft tissue phenotype, bone density, occlusal forces, and available interocclusal space), emphasizing that future studies should focus on these parameters.

The limitation of the present study may be that only the available literature across databases was considered, rather than all possible studies.

## 5. Conclusions

Based on the present scoping review, a set of fundamental parameters critical for the design of hybrid abutments has been identified: transmucosal and prosthodontic height of implant abutments. Representative values were established according to published data and clinical recommendations (>2 mm for each parameter). These parameters will serve as the foundation for constructing an initial parametric model to be used in generative design. This foundational model will be further developed and expanded based on ongoing literature insights, future experimental findings, and established knowledge from structural analysis and mechanical stability assessments.

In addition, the present study provides a comprehensive synthesis of existing evidence on the influence of abutment design parameters, helping to identify gaps in current research and guiding future investigations. By consolidating findings from multiple sources, the review offers suggestions for clinicians to invest more time and thought in selecting abutment designs to optimize peri-implant tissue health and mechanical performance. Furthermore, the study establishes a structured framework that can support development of more predictive and patient-specific implant abutment designs, ultimately contributing to improved clinical outcomes and advancing the field of implant prosthodontics.

## 6. Future Directions

In the future, studies on transmucosal and prosthodontic height of implant abutments will likely move toward more personalized designs that take into account each patient’s soft tissue thickness and aesthetic needs. Generative design in implant prosthodontics remains in its developmental phase, with definitive technical solutions and standardized clinical guidelines yet to be established. However, due to industry involvement and interest, it is expected to reach technical readiness in a short period of time. Using generative design to customize hybrid implant abutments could reshape treatment planning by making it easier to develop solutions that are both biologically compatible and functionally effective for prosthodontic use.

## Figures and Tables

**Table 1:** Studies included in the review dealing with transmucosal height of implant abutments.

Authors, Year	Title	Type of the Study	Sample Size	Conclusion
Misch et al. (2025) [29]	Combined Effect of Abutment Height and Restoration Emergence Angle on Peri-Implant Bone Loss Progression: A Retrospective Analysis	Retrospective study	192 implants 119 patients	Abutment height > 2 mm may play a role in reducing peri-implantitis and marginal bone loss
Galindo-Moreno et al. (2022) [30]	Limited marginal bone loss in implant-supported fixed full-arch rehabilitations after 5 years of follow-up	Retrospective study	160 implants 19 patients	Implants with transmucosal abutments longer than 2 mm show less than 0.5 mm from the implant shoulder to the marginal bone
Gehrke et al. (2025) [31]	Effects of Different Patient and Prosthetic Variables on Marginal Bone Behavior in Dental Implants: A Clinical Retrospective Study	Retrospective study	111 implants 50 patients	Transmucosal abutment height (>2.5 mm) was associated with reduced bone loss

**Table 2:** Studies included in the review dealing with prosthodontic height of implant abutments.

Authors, Year	Title	Type of the Study	Sample Size	Conclusion
Quispe-López et al. (2024) [32]	The influence of implant depth, abutment height and mucosal phenotype on peri-implant bone levels: A 2-year clinical trial	Clinical trial	71 implants 26 patients	Long abutments (>2 mm) result in the lowest level of marginal bone loss
Quintas-Hijós et al. (2025) [33]	Influence of intermediate abutment height and timing of placement on marginal bone loss in single implant-supported crowns: a 12-month follow-up randomized clinical trial	Randomized clinical trial	60 implants 54 patients	Immediate abutments of 2–3 mm resulted in lower marginal bone loss compared to 1.5 mm immediate abutments or any delayed abutments
Tajti et al. (2024) [34]	Less marginal bone loss around bone-level implants restored with long abutments: A systematic review and meta-analysis	Systematic review and meta-analysis	/	It can be tentatively concluded that longer abutments for bone-level implants appear to be a favorable treatment option for reducing early marginal bone loss
Ríos-Santos et al. (2020) [35]	One Abutment One Time: A Multicenter, Prospective, Controlled, Randomized Study	Controlled randomized study	231 implants 147 patients	Height of the prosthetic abutment has a statistically significant influence on bone loss, concluding that there is greater peri-implant bone loss using 1 mm high abutments compared to using 2 mm high abutments
Arai et al. (2025) [36]	Marginal bone loss in dental implants: A literature review of risk factors and treatment strategies for prevention	Literature review	/	Abutment with a height of at least 2 mm should be selected
Galindo-Moreno et al. (2022) [37]	Early marginal bone loss around dental implants to define success in implant dentistry: A retrospective study	Retrospective study	590 implants 176 patients	A minimum of 2 mm abutment height is recommended to get adequate protection against peri-implant bone resorption
Alseddiek et al. (2023) [38]	Retention of hybrid-abutment-crowns with offset implant placement: influence of Crown materials and Ti-base height	In vitro study	42 hybrid-abutment crowns	The retention of the three different hybrid-abutment crown types is greatly improved by the height of the abutment evaluated
Müller et al. (2021) [39]	Impact of cement type and abutment height on pull-off force of zirconia reinforced lithium silicate crowns on titanium implant stock abutments: an in vitro study	In vitro study	64 abutments	The abutment height had a subordinate impact
Song et al. (2021) [40]	Fracture strength analysis of titanium insert-reinforced zirconia abutments according to the axial height of the titanium insert with an internal connection	In vitro study	60 implants	Axial height of the titanium insert should be designed not to be less than 3 mm to increase the fracture strength and promote the long-term stability of implant-supported fixed prostheses with titanium insert-reinforced zirconia abutments
Hendi et al. (2024) [41]	The effect of different implant—abutment types and heights on screw loosening in cases with increased crown height space	In vitro study	64 abutments	4 mm post-height abutment is more effective than 7 or 10 mm in maintaining screw torque

## Data Availability

The original contributions of this study are detailed within the article. For additional information or clarifications, interested readers are encouraged to contact the corresponding author.
